# Co-administration of vitamin D3 and *Lacticaseibacillus paracasei* DG increase 25-hydroxyvitamin D serum levels in mice

**DOI:** 10.1186/s13213-021-01655-3

**Published:** 2021-10-18

**Authors:** Ignazio Castagliuolo, Melania Scarpa, Paola Brun, Giulia Bernabe, Valeria Sagheddu, Marina Elli, Walter Fiore, Valerio De Vitis, Simone Guglielmetti

**Affiliations:** 1 Department of Molecular Medicine, University of Padua, 35121 Padua, Italy; 2 AAT-Advanced Analytical Technologies S.r.l., Fiorenzuola d’Arda, Piacenza, Italy; 3 SOFAR S.p.A., Trezzano Rosa, Milan, Italy; 4 Division of Food Microbiology and Bioprocesses, Department of Food, Environmental and Nutritional Sciences (DeFENS), University of Milan, 20133 Milan, Italy

**Keywords:** Probiotics, Vitamin D, Cholecalciferol supplementation, Bioavailability, Biosurfactant

## Abstract

**Purpose:**

Subclinical vitamin D (vitD) deficiency enhances the predisposition to a myriad of acute and chronic pathologies in many people worldwide. Due to the scarcity of vitD-rich foods, the consumption of supplements or fortified foods can be required to maintain healthy serum levels of 25-hydroxyvitamin D [25(OH)D], and the major circulating form of vitD that is commonly measured in serum to determine the vitD status. Since the vitD absorption seems to resemble that of lipids, improved emulsification in the gut could favor vitD permeation through the enterocyte membrane. Contextually, we hypothesized that a microorganism with cholecalciferol (vitD3)-solubilization properties may potentially result in enhanced serum vitD levels.

**Methods and results:**

Six probiotic strains were screened for their ability to create a stable suspension of vitD3 in water: *Lacticaseibacillus paracasei* DG, *L. paracasei* LPC-S01, *L. paracasei* Shirota, *L. rhamnosus* GG, *Limosilactobacillus reuteri* DSM 17938, and *Lactobacillus acidophilus* LA5. The DG strain displayed the strongest vitD3 solubilization ability and, consequently, were used in an in vivo trial where a commercial preparation of vitD3 in refined olive oil was administered by gavage to CD-1 mice with or without the concurrent administration of *L. paracasei* DG. ELISA measurements showed that the DG strain significantly increased the serum levels of 25(OH) D when administered once a day for 1 week in association with the vitD3 supplement.

**Conclusion:**

This preliminary pre-clinical study suggests that the combined administration of *L. paracasei* DG with an oil-based cholecalciferol supplement could contribute to the maintenance of the adequate 25(OH) D serum levels in people at risk of vitD deficiency.

## Background

Vitamin D is the generic name for a group of liposoluble pro-hormones consisting of five different forms of secosteroids named D1, D2, D3, D4, and D5. In humans, the most important forms are vitamin D2 (also known as ergocalciferol), which has vegetal origin, and vitamin D3 (cholecalciferol), which derives from animal food or is synthesized in the skin upon sun exposure. Vitamins D2 and D3 are in a biologically inactive form and must undergo two hydroxylation reactions, the first in the liver and the second in the kidneys, to be converted in the bioactive forms. Specifically, in the liver, vitamins D2 and D3 are converted into 25-hydroxycholecalciferol (calcifediol) and 25-hydroxyergocalciferol. Calcifediol and 25-hydroxyergocalciferol are indicated together as 25-hydroxyvitamin D (25(OH)D), which is the major circulating form of vitamin D and is commonly measured in serum to determine human vitamin D status (Hollis 1996). Finally, the bioactive form of vitamin D is produced in the kidneys, where calcifediol is further hydroxylated into calcitriol (1,25-dihydroxycholecalciferol; 1,25(OH)2D3), and 25-hydroxyergocalciferol into 1,25-dihydroxyergocalciferol (1,25(OH)2D2) (Holick et al. 1971, Norman et al. 1971).

Vitamin D has been demonstrated to be essential for multiple biological processes including bone–mineral homeostasis, regulation of innate and adaptive immune function, cellular proliferation and differentiation, and apoptosis (Umar et al. 2018). Accordingly, numerous studies demonstrated a relationship between vitamin D deficiency and increased risk of a myriad of acute and chronic pathologies such as cardiovascular disease, cancer, autoimmune diseases, infectious diseases, and neurological conditions (Holick 2017, Yang et al. 2013, Zisi et al. 2019).

It was estimated that vitamin D deficiency/insufficiency affects about one billion people, being one of the most underdiagnosed and undertreated medical condition worldwide (Holick 2007, Holick 2017). Numerous population groups are at increased risk of vitamin D deficiency and insufficiency, including pregnant women, people with increased skin melanin pigmentation, obese children and adults, and persons with abstinence from direct sun exposure (Holick 2017). In addition, vitamin D deficiency can also be determined by therapies such as those directed to the reduction of blood cholesterol levels, which can reduce the absorption of sterols and, therefore, also the absorption of fat-soluble vitamins, including vitamin D (Nasirpour et al. 2019). Considering the scarcity of foods rich in vitamin D, the integration of diet with reinforced foods and supplements of vitamins D2 and D3 is attracting increasing attention as an effective strategy for several health-promoting purposes including the prevention of fractures in elderly women (Bergman et al. 2010), the reduction of gestational diabetes, low birth weight, and preeclampsia in pregnancy (Palacios et al. 2019); the anti-inflammatory activity in the lungs of mechanically ventilated patients (Leclair et al. 2019); the improvement of lipid profiles in subjects with hypercholesterolemia (Dibaba 2019) and chronic kidney disease (Milajerdi et al. 2019); and the reduction of atopic dermatitis severity (Hattangdi-Haridas et al. 2019). Notably, it was also suggested that vitamin D supplementation can significantly contribute to the prevention and treatment of the COVID-19 (Boulkrane et al. 2020, Cutolo et al. 2020, Grant et al. 2020).

The proportion of ingested vitamin D amount that ultimately reaches the systemic circulation can vary abundantly, depending on a plethora of factors such as the molecular form of the ingested vitamin, the matrix of co-ingested food (e.g., the amount and type of fatty acids and the presence of dietary fibers), the interaction with other fat soluble compounds, the bile secretions, the intestinal wall integrity, the gastro-intestinal luminal pH, and host-associated factors (e.g., age, inflammatory state, obesity). Most of these factors influence the possibility that the non-polar lipid molecules of vitamin D can get accessed to enterocytes and get absorbed (Maurya and Aggarwal 2017). It is generally assumed that vitamin D absorption follows the same steps described for major lipids such as triacylglycerols, cholesterol, and phospholipids, i.e., emulsification and solubilization in mixed micelles, diffusion in the watery luminal environment, and permeation through the enterocyte membrane (Borel et al. 2015; Tso and Fujimoto 1991). This passive diffusion process through mixed micelles is probably not the exclusive mechanism of absorption but appears to be the dominant mechanism for the non-hydroxylated species of vitamin D (i.e., vitamin D2 and vitamin D3) at pharmacological concentrations (Borel et al. 2015, Maurya and Aggarwal 2017). In light of the proposed mechanism of vitamin D absorption, most commercial vitamin D supplements are constituted by fat-soluble preparations. In addition, supplements including vitamin D3 nanoemulsion formulations, which have been demonstrated to enhance cholecalciferol bioavailability, have been marketed (Marwaha and Dabas 2019). Interestingly, it was also reported a significant increase of serum 25(OH) D in hypercholesterolemic adults in response to the oral administration of the probiotic bacterial strain *Limosilactobacillus reuteri* (formerly *Lactobacillus reuteri*) NCIMB 30242 (Jones et al. 2013). Notably, vitamin D was the only fat-soluble vitamin affected by the probiotic intake, whereas the levels of vitamins A, E, and beta-carotenes did not change (Jones et al. 2013). Since it was reported that dietary vitamin D absorption could be enhanced at higher hydrogen ion concentrations (Hollander et al. 1978), the authors hypothesized that the observed increased serum levels of vitamin D could be a consequence of the acidification of the small bowel lumen resulting from the metabolism of *L. reuteri*. Despite the promising results, to the best of our knowledge, no other studies investigated the potential ability of probiotic microorganisms to improve vitamin D absorption and bioavailability. For this reason, in the present study, we aimed to test in vivo in a murine model the ability of a probiotic bacterial strain to increase the serum levels of vitamin D, when the bacterial cells were administered together with a commercial preparation of vitamin D3 in olive oil. We show that the probiotic bacterial strain used in the study, which was selected based on its increased ability to solubilize cholecalciferol compared to other Lactobacillaceae probiotics, can significantly increase the serum levels of 25(OH) D in mice following administration once a day for 1 week in association with the commercial vitamin D3 supplement.

## Results

### *Lacticaseibacillus paracasei* DG cells efficiently emulsify cholecalciferol in water

**Fig. 1:**
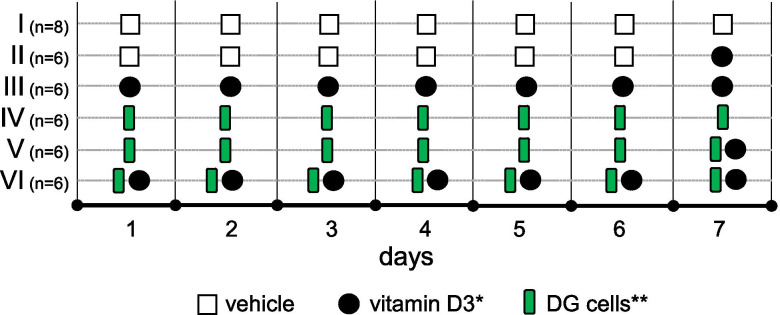
Cholecalciferol-emulsification properties of Lactobacillaceae probiotic strains. Statistically significant differences were determined through unpaired t test with Welch’s correction (*n*=3) performed after the Levene’s test, which evidenced that the variance between groups was different (Levene’s *P*= 0.005). **P<0.01; *P<0.05

Six lactic acid bacterial strains were tested for their potential ability to create a stable suspension of vitamin D in water: *L. paracasei* DG, *L. paracasei* LPC-S01, *L. paracasei* Shirota, *L. rhamnosus* GG, *L. reuteri* DSM 17938, and *L. acidophilus* LA5. To this aim, PBS-washed bacterial cells in PBS were mixed with a solution of vitamin D3 in refined olive oil (10:1 v/v). Then, an aliquot of the phase below the oil was analyzed by HPLC to quantify the solubilization of cholecalciferol in the aqueous fraction. The obtained results revealed a significant increase of cholecalciferol in the aqueous phase only for strains *L. paracasei* DG and *L. rhamnosus* GG (Fig. 1). In particular, the cells of *L. paracasei* DG displayed the strongest ability over the other strains tested. Interesting, this ability resulted to be strain-specific, since the vitamin D3 solubilization ability of the other two strains of the same species, i.e., *L. paracasei*, LPC-S01, and Shirota, resulted negligible (Fig. 1). According to the results of this experiment, *L. paracasei* DG was selected for the in vivo vitamin D bioavailability experiment in mouse model.

### Dietary vitamin D increases serum 25(OH) D when co-administered with probiotic bacterial cells

**Fig. 2:**
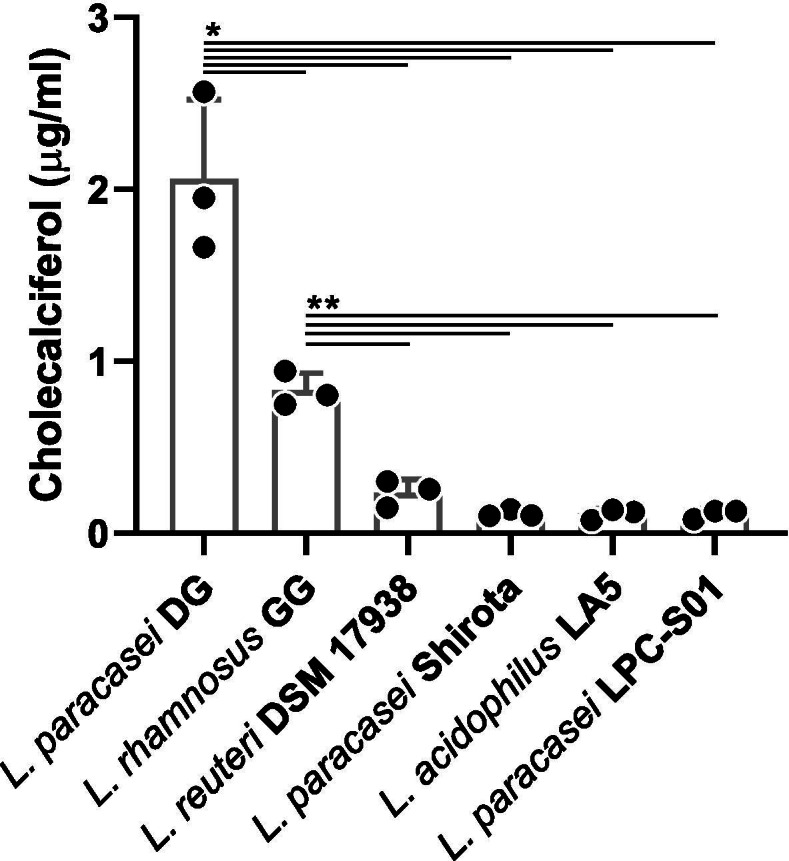
Design of the trial in mouse model. Vehicle, vitamin D3, and L. *paracasei* DG cells were administered to mice by gavage. Vehicle consisted of an aqueous solution of sucrose 20% + glycerol 10% (w/vol). Bacterial cells were suspended in the same vehicle. *500 IU of vitamin D3 in refined olive oil (DIBASE) were administered per single gavage. **10^8^ CFUs of L. *paracasei* DG were administered per single gavage

In order to assess the ability of the probiotic bacterium *L. paracasei* DG to enhance vitamin D bioavailability, a commercial preparation of cholecalciferol in refined olive oil (DIBASE) was administered to mice by gavage with or without the concurrent administration of *L. paracasei* DG cells, according to the study design described in Fig. 2. Then, the concentration of 25(OH) D was measured by ELISA in mouse serum collected 3 h after the last gavage.

**Fig. 3:**
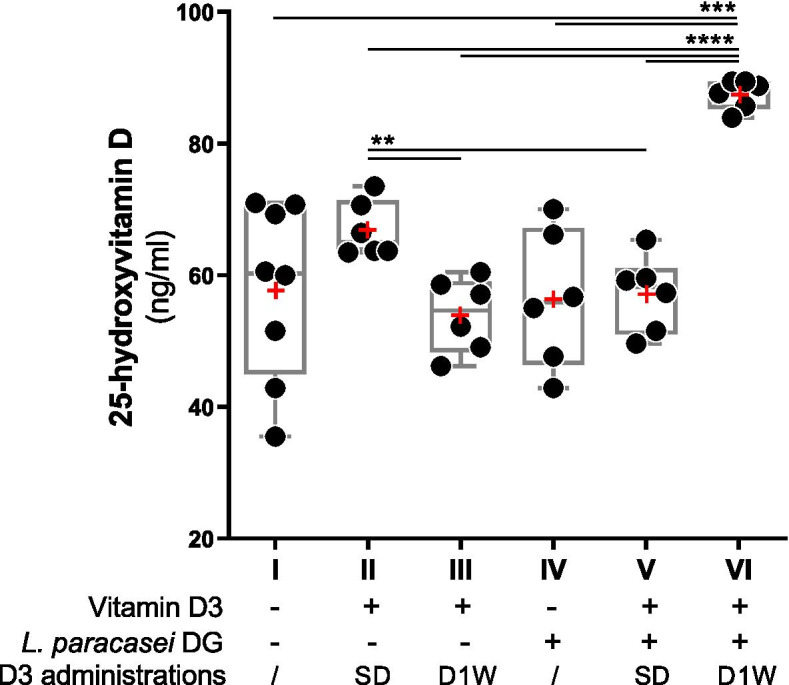
Levels of 25-hydroxyvitamin D [25(OH)D] in mouse serum samples collected 3 h after the last gavage, as determined through ELISA measurements. Red symbols (+) indicate the mean. *Lacticaseibacillus paracasei* DG cells were administered to mice by gavage once a day for 1 week. Sample groups (from I to VI) are according to Fig. 2. − not administered; + administered. SD, single-dose administration of vitamin D3; D1W, daily administration of vitamin D3 for 1 week. Statistically significant differences were determined through unpaired t test with Welch’s correction (*n*=3) performed after the Levene’s test, which evidenced that the variance between groups was different (Levene’s *P*= 0.021). ****P<0.0001; ***P<0.001; **P<0.01

In control CD-1 mice on regular chow diet (mouse group I), 25(OH) D serum levels ranged between 36 and 71 ng/ml (mean ± standard deviation 58 ± 13 ng/ml; Fig. 3). A single-dose (group II) or daily 1 week (D1W; group III) administration of vitamin D3 had no significant effect on serum 25(OH) D compared to the control (Fig. 3). Similarly, daily administration of the probiotic *L. paracasei* DG for a week, alone or in combination with a single dose of vitamin D3, did not affect significantly 25(OH) D serum levels compared to control mice (Fig. 3). Nonetheless, serum 25(OH) D level was slightly but significantly higher after the single-dose supplementation of vitamin D3 (group II; 67 ± 4 ng/ml) compared to D1W vitamin D3 (group III; 54 ± 6 ng/ml) and single-dose vitamin D3 + DG cells (group V; 57 ± 6 ng/ml) administrations (Fig. 3). Collectively, the serum levels of 25-hydroxyvitamin D for mice in groups I to V were all between 35 and 74 ng/ml (58 ± 10 ng/ml). On the contrary, when 10^8^ CFUs of *L. paracasei* DG were administered in combination with the vitamin D3 supplement once a day for 1 week, the serum concentration of 25(OH) D ranged between 84 and 89 ng/ml (group VI; 88 ± 2 ng/ml) (Fig. 3), corresponding on average to 50, 62, and 55% increase compared to control (group I), 1-week administration of vitamin D3 (group III) and the sole administration of DG cells (group IV), respectively. Notably, the significant increase of 25(OH) D serum levels was observed only when *L. paracasei* DG cells were administered together with vit. D3 for 1 week and not when the vitamin was administered as a single dose at day 7.

## Discussion

Severe vitamin D deficiency is very rare in developed countries. Nonetheless, increasing evidence is demonstrating that subclinical vitamin D deficiency affects a large number of people worldwide, resulting in an enhanced predisposition to several medical conditions ranging from frailty (Halfon et al. 2015) to neurological diseases (Dobson et al. 2018). For this reason, in recent years, several experts advised that consumption of supplements or fortified foods is required to meet the daily need of vitamin D (Papadimitriou 2017; Smith et al. 2017; Wimalawansa 2012). However, the precise extent of supplementation needed is difficult to be defined, due to intrinsic differences in the requirement of any specific population group and also because of the wide inter-subject variability in vitamin D absorption efficiency (Borel et al. 2015).

Although with an efficiency lower than that of triacylglycerols, it is generally assumed that the absorption of vitamin D resembles that of lipids in the human small bowel (Borel et al. 2015). Therefore, it appears plausible that improved emulsification of vitamin D in the gut may favor its diffusion across the watery environment of lumen, enhancing the contact with the apical side of the enterocytes, and, therefore, permitting its permeation through the enterocyte membrane.

Several species of the Lactobacillaceae family have been demonstrated to produce biosurfactants (Satpute et al. 2016), i.e., amphiphilic molecules of different chemical nature (e.g., glycolipids, phospholipids, lipopeptides, and other polymers) that may have deterging, emulsifying, foaming, or dispersing activity (Jahan et al. 2020; Santos et al. 2016). Therefore, it can be hypothesized that biosurfactant-producing microorganisms, once in the gut, may contribute to the emulsification of vitamin D, favoring its contact with the epithelium and, consequently, its absorption. Interestingly, biosurfactant production has been also reported for microorganisms that are commonly used as probiotics such as *L. casei/paracasei*, *L. rhamnosus*, and *L. acidophilus* (Morovic et al. 2016). Contextually, in this study, we selected six Lactobacillaceae strains with well-documented probiotic properties to test in vitro their ability to solubilize cholecalciferol in water. Specifically, we included in the analysis *Lacticaseibacillus paracasei* Shirota, which is considered the first probiotic ever (Nanno et al. 2011), *Lacticaseibacillus rhamnosus* GG, the most studied probiotic bacterium (Capurso 2019), and *Lacticaseibacillus paracasei* DG, one of the most commonly consumed probiotics in Italy (Arioli et al. 2018; Cremon et al. 2018). In addition, we also studied three other probiotic strains with documented properties that can be commonly found in probiotic foods and supplements marketed in Europe, i.e., *Lactobacillus acidophilus* LA5 (Bogovic Matijasic et al. 2016; Zarrati et al. 2013), *Lacticaseibacillus paracasei* LPC-S01 (Balzaretti et al. 2015; Koirala et al. 2020), and *Limosilactobacillus reuteri* DSM 17938 (Mobini et al. 2017; Patro-Golab and Szajewska 2019). The experiment we carried out revealed a wide difference among tested bacteria, which displayed vitamin D3-emulsification ability in a strain-specific fashion. Interestingly, only one out of the three *L. paracasei* tested, viz. strain DG, significantly increased the amount of cholecalciferol in the aqueous phase. The emulsifying capacity of *L. paracasei* DG was also significantly higher than that of *L. rhamnosus* GG, which was previously demonstrated to produce biosurfactant molecules (Ciandrini et al. 2016). The molecular determinant of the emulsifying ability of strain DG is not known, but we can speculate that a significant contribution can be provided by the unique rhamnose-rich hetero-exopolysaccharide (EPS) that *L. paracasei* DG accumulates on the outer cell surface (Balzaretti et al. 2017). Reportedly, bacterial EPS macromolecules may exert efficient emulsifying properties toward different hydrophobic molecules such as crude oils and hydrocarbons (Calvo et al. 2002; Gutierrez et al. 2009; Gutierrez et al. 2013).

Due to the peculiar ability of DG cells to facilitate the solubilization of cholecalciferol in an aqueous environment, this strain was administered to mice in association with the vitamin D3 supplement used in the in vitro solubilization assay, in order to assess the potential increase of vitamin bioavailability.

The serum levels of 25-hydroxyvitamin D in CD-1 male mice were not significantly affected by the administration of vitamin D3 alone, either in a single dose or daily for 1 week. Similarly, we did not observe a significant change of 25(OH) D levels when the probiotic bacterium was gavaged daily for 1 week, either alone or together with a single dose of vitamin D3 administered the last day of the trial. For all these five groups of mice, in fact, the levels of 25-hydroxyvitamin D were in a range that is in line with the literature concerning the normal vitamin D levels of mice under regular chow diet (Ghaly et al. 2018, Mallya et al. 2016). Nonetheless, notably, when the vitamin D3 supplement and the probiotic bacterial cells were mixed and administered for 7 consecutive days, we observed a sharp enhancement of serum 25-hydroxyvitamin D levels in all mice, suggesting the potential ability of the bacterial cells to enhance the bioavailability, in accordance with our initial hypothesis. However, the mechanism supporting the increase in 25(OH) D levels following the co-administration of vitamin D with strain DG is not known, and additional experiments are needed to confirm that the cholecalciferol solubilization properties of DG cells may be relevant. Other possible mechanisms can be hypothesized, such as the acidification of the luminal pH due to lactic acid production, as previously proposed (Jones et al. 2013). In addition, we can speculate that gavaged bacterial cells may stimulate the secretion of bile and pancreatic juices, which could result into more efficient micelle formation and, consequently, enhanced cholecalciferol absorption. In support of this speculation, a recent publication showed that *L. paracasei* DG administration increased the amino acid absorption from plant proteins in physically active men (Jager et al. 2020).

Vitamin D was shown to exert important roles in the gut, where it is involved in the modulation of both mucosal immunity and normal growth of epithelial cells (Cross et al. 2011). In addition, vitamin D status was associated with the composition and function of the intestinal microbiota (Weiss and Litonjua 2015). Therefore, the spectrum of actions of vitamin D in the gut resembles the activities that have been commonly reported for probiotic microorganisms (Shang and Sun 2017), and for this reason, the co-administration of probiotics and vitamin D was supposed to synergistically promote beneficial effects for the human intestinal health (Shang and Sun 2017). In this context, the combination of vitamin D3 with *L. paracasei* DG appears particularly promising, considering the demonstrated probiotic properties of this bacterium, which include the ability to efficiently survive the gastrointestinal transit (Radicioni et al. 2019; Arioli et al. 2018), modulate the gut microbiota composition and butyrate levels in healthy adults (Ferrario et al. 2014) and irritable bowel syndrome (IBS) patients (Cremon et al. 2018), and regulate the immune response in vitro (Balzaretti et al. 2015; Balzaretti et al. 2017), ex vivo (Compare et al. 2017), and in vivo (D’Inca et al. 2011).

## Conclusion

The results of this preliminary pre-clinical study suggest that the combined administration of *L. paracasei* DG cells with a conventional supplement of cholecalciferol may contribute to the maintenance of the adequate 25-hydroxyvitamin D serum levels in the population groups at risk of vitamin D deficiency. Further studies are warranted to assess synergistic effects of the combination between vitamin D and DG cells in terms of immune response, intestinal mucosal homeostasis, and microbiota composition and function, which can confer health benefits both at intestinal mucosal level and systemically.

## Methods

### Bacterial strains and cultivation conditions

Six bacterial strains belonging to the Lactobacillaceae family were investigated: *Lacticaseibacillus paracasei* (formerly *Lactobacillus paracasei*) CNCM I-1572 (L. casei DG®; Enterolactis®, SOFAR S.p.A.), *Lacticaseibacillus paracasei* DSM 26760 (strain LPC-S01), *Lacticaseibacillus paracasei* Shirota, *Lacticaseibacillus rhamnosus* (formerly *Lactobacillus rhamnosus*) ATCC 53103 (strain GG), *Limosilactobacillus reuteri* DSM 17938, and *Lactobacillus acidophilus* DSM13241 (strain LA5). All strains were cultivated in de Man Rogosa Sharpe (MRS; Difco) broth at 37°C overnight.

### In vitro evaluation of vitamin D3 emulsification by probiotic bacterial cells

The cell pellet was recovered from the broth culture by centrifugation, washed twice with phosphate buffer saline solution (PBS; pH 7.3), and resuspended in PBS to an OD_600nm_ = 5. Then, 100 μl of DIBASE (50.000 IU/2.5 ml cholecalciferol in refined olive oil, Abiogen Pharma, Italy), corresponding to 2000 IU (2.5 μg) of cholecalciferol was dispersed in 1 ml of the bacterial suspension and incubated under magnetic stirring (500 rpm) for 45 min. Next, after mild centrifugation (2000 rpm, 2 min), 400 μl of the aqueous phase laying below the oil phase was taken by means of the needle of a sterile syringe. Subsequently, cholecalciferol was extracted from the aqueous samples using the following method: 400 μl of methanol was added to the sample and the mixture was extracted once with two volumes of hexane. The hexane phase obtained after centrifugation (13000 rpm, 3 min) was evaporated to dryness under nitrogen, and the dried residue was dissolved in 150 μl of HPLC mobile phase. A volume of 100 μl was used for HPLC analysis.

### HPLC analysis

Cholecalciferol was separated using a 4.6 × 100 mm, 2.7 μm Poroshell 120 EC-C18 column in a HPLC system comprising an Agilent separation module (1260 infinity II pumps with autosampler), and 1260 infinity II variable wavelength detector (detection at 265nm for vitamin D). The chromatographic protocol was according to Goncalves et al. (2011). In brief, the mobile phase was 60% acetonitrile, 38% methanol, and 2% water. Flow rate was 1 ml/min, and the column was kept at a constant temperature (40°C). Cholecalciferol was identified by spectral analysis and/or retention time and co-injection compared with pure standard. Quantification was performed with open Lab HPLC agilent software comparing peak area with standard reference curve.

### Mice and treatments

Male CD-1 mice (8 weeks old) were purchased from Charles River (Monza, Italy). Mice were housed in group of 4 per cage and maintained in a temperature-controlled environment (22 ± 2°C) under a 12-h-light/dark cycle with regular chow food and tap water provided ad libitum. Following a week of acclimation period, they were randomly assigned to one of six experimental groups (Fig. 2). Each animal received the specific treatment at 9 a.m. by intragastric gavage (24 gauge, 9-cm catheter) in a total volume of 100 μl. Mice receiving vitamin D3 were gavaged with 10,000 IU of vitamin D3 in refined olive oil (DIBASE preparation) per kilogram of body weight either as a single dose or daily for a week. To this purpose, 25 μl aliquots, each containing 500 IU of vitamin, were prepared in sterile conditions for supplementation to the animals, having an average body weight of 50 g, and stored at room temperature. Mice receiving the probiotic cells were gavaged with 10^8^ CFUs of *L. paracasei* DG in a single administration per day for a week. To this purpose, bacterial broth culture was centrifuged, and the resulting cell pellet was washed twice with sterile saline solution and finally resuspended with the appropriate volume of an aqueous solution of sucrose 20% + glycerol 10% (w/vol). The suspension was diluted to OD_600nm_ = 1 and then partitioned into 150 μl aliquots, which were immediately frozen at −20°C until dry ice delivery at the Department of Molecular Medicine of the University of Padova. Each aliquot had a cell concentration of 1.6×10^9^ CFU/ml (corresponding to 2.4×10^8^ CFU/aliquot) as calculated by agar plate count on MRS agar. When administered together (groups V and VI), the aliquot of vitamin D and the bacterial cell suspension were mixed by manual shaking and pipetting immediately before gavage. Control mice (group I) received only the aqueous solution of sucrose 20% + glycerol 10% (w/vol) for 1 week.

### Vitamin D quantification in mouse serum

Three hours after the last treatment, mice were anesthetized with halothane and the blood was collected by cardiac puncture. Serum was separated from blood through centrifugation, collected, and immediately frozen until the dosage of the vitamin D. The serum level vitamin D was quantified in the form of 25-hydroxyvitamin D (25(OH)D) by a competitive binding assay (25OH vitamin D total ELISA, Gentaur, Bergamo, IT) optimized for mouse serum. Vitamin D3 bioavailability was tested through ELISA quantification of the 25(OH) D in mouse serum, because the blood level of this metabolite is conventionally considered a valid biomarker of the vitamin D status (Thienpont et al. 2012).

### Statistical analysis

Data are reported as the mean ± standard deviation. Data were analyzed with the Levene’s test for assessing the equality of variances. Then, since the variance between groups resulted different, we performed the Welch’s ANOVA test followed by an unpaired *t* test with Welch’s correction to find statistically significant differences between groups. Differences were considered significant at *P* < 0.05.

## Data Availability

The datasets used and/or analyzed during the current study are available from the corresponding author on reasonable request.
